# Long-term outcomes of liver transplantation in patients with hepatitis C infection are not affected by HCV positivity of a donor

**DOI:** 10.1186/s12876-016-0551-z

**Published:** 2016-11-15

**Authors:** Maria Stepanova, Mehmet Sayiner, Leyla de Avila, Zahra Younoszai, Andrei Racila, Zobair M. Younossi

**Affiliations:** 1 Betty and Guy Beatty Center for Integrated Research, Inova Health System, Claude Moore Health Education and Research Building 3300 Gallows Road, Falls Church, VA 22042 USA; 2 Center for Outcomes Research in Liver Diseases, Washington, DC USA; 3 Center for Liver Diseases, Department of Medicine, Inova Fairfax Hospital, Falls Church, VA USA

**Keywords:** Liver transplant, HCV, Extended criteria donor

## Abstract

**Background:**

The use of HCV-positive livers for HCV-positive recipients is becoming more common. Our aim is to evaluate long-term outcomes in liver transplant recipients transplanted with HCV antibody-positive organs.

**Methods:**

From the Scientific Registry of Transplant Recipients (1995–2013), we selected all adult liver transplant recipients with HCV, and cross-sectionally compared long-term graft loss and mortality rates between those who were transplanted from HCV antibody-positive (HCV+) vs. HCV antibody-negative donors.

**Results:**

We included 33,668 HCV+ liver transplant recipients (54.0 ± 7.7 years old, 74.1% male, 71.0% white, 23.6% with liver malignancy). Of those, 5.7% (*N* = 1930) were transplanted from HCV+ donors; the proportion gradually increased from 2.9% in 1995 to 9.4% in 2013. Patients who were transplanted from HCV+ positive donors were more likely to be discharged alive after transplantation (95.4% vs. 93.9%, *p* = 0.006), but this difference was completely accounted for by a greater proportion of HCV+ donors in more recent study years (*p* = 0.10 after adjustment for the transplant year). After transplantation, both mortality in HCV patients transplanted from HCV+ donors (12.5% in 1 year, 24.2% in 3 years, 33.0% in 5 years) and the graft loss rate (2.2% in 1 year, 4.8% in 3 years, 7.5% in 5 years) were similar to those in HCV patients transplanted from HCV-negative donors (all *p* > 0.05).

**Conclusions:**

Over the past two decades, the use of HCV+ organs for liver transplantation has tripled. Despite this, the long-term outcomes of HCV+ liver transplant recipients transplanted from HCV+ donors were not different from those who were transplanted with HCV-negative organs.

**Electronic supplementary material:**

The online version of this article (doi:10.1186/s12876-016-0551-z) contains supplementary material, which is available to authorized users.

## Background

Chronic hepatitis C infection is currently the most common indication for liver transplantation in the U.S. [1]. After the disease progresses to its most advanced stages which include hepatocellular carcinoma and liver failure, the only alternative to liver-related death is receiving a liver transplant. Since the progression rate increases with age [2], and given that the highest prevalence of HCV infection in the U.S. has been reported in the baby boomer population [3] who are currently between 50 and 70 years of age, the need for liver transplantation in HCV-infected patients is likely to remain high in the coming years. It is unclear whether the recently approved direct-acting antiviral (DAA) regimens for HCV, for which their high efficacy and minimal contraindications are at this point counter-balanced by access-related barriers [4–6], are able to reverse this trend at the national level any time soon.

Due to the shortage of organs, the use of HCV-infected grafts has long been considered as a potentially viable alternative for patients who are already infected with HCV. Although concerns about the presence of HCV-related damage to the graft and about viral transmission initially seemed plausible, in the last two decades, an increasing number of centers have adopted the practice of extending their criteria to include HCV-positive donors for using in HCV-positive recipients. As of now, the results reported from both single-center and multi-center studies have been largely consistent about the lack of an additional risk to a recipient associated with the presence of HCV infection in a graft, given that both a recipient and a donor meet other selection criteria [7–11]. It is important to note that most of these studies had a relatively short follow-up. On the other hand, the results reported from other multi-center studies have suggested that the use of HCV-positive donors is not truly risk-free and may, in fact, result in an additional clinical burden which includes an increased risk of developing advanced post-transplant fibrosis and more rapid recurrence of hepatitis in the graft [12, 13]. It is unclear whether this burden could eventually translate into compromised long-term outcomes with long enough follow-up.

The aim of this study is to report post-transplant outcomes in HCV-positive recipients transplanted from HCV-positive donors, and to evaluate the risk for mortality and graft loss associated with the use of HCV-positive donors, using the most recent long-term follow-up data from a nationwide registry of liver transplant recipients.

## Methods

### Study cohort

This study used data from the Scientific Registry of Transplant Recipients (SRTR). The SRTR data system includes data on all donor, wait-listed candidates, and transplant recipients in the U.S., submitted by the members of the Organ Procurement and Transplantation Network (OPTN), and has been described elsewhere. The Health Resources and Services Administration (HRSA), U.S. Department of Health and Human Services provides oversight to the activities of the OPTN and SRTR contractors.

In this study, we included all liver transplant recipients with HCV of at least 18 years of age and older who underwent liver transplantation between 1995 and 2013 in the U.S. In both recipients and donors, their anti-HCV serology status was used to define those with and without HCV (HCV+ and HCV-, respectively). No HCV RNA testing was reported in SRTR.

The length of hospital stay for patients following liver transplantation was calculated in days from the date of transplant to the date of discharge. Inpatient mortality and history of acute rejection during inpatient stay were recorded. We also collected information on organ donors that included high risk donors classified by the CDC criteria, basic demographics and clinical history (diabetes, cancer), and recorded procurement from a non-heart beating donor.

The primary outcomes included in this study were inpatient mortality, post-transplant mortality (calculated by matching with the Social Security Death Master File, provided by SRTR), and post-transplant graft failure (defined as a documented re-transplant or a graft loss-related cause of death). Patients undergoing re-transplantation were included in the mortality analysis with their most recent transplants only.

Liver transplant recipients discharged alive were followed at 6 and 12 months post-transplant, and then annually until death, re-transplantation, or loss to follow-up. Patients with no documented date of death were presumed alive as of September 1, 2015. The time to events for survival analysis was calculated from the date of transplant.

### Statistical analysis

The clinico-demographic parameters and outcomes of liver transplant recipients with HCV+ were compared between those transplanted from HCV+ and HCV- donors using a Kruskall-Wallis non-parametric test and a chi-square test for homogeneity. The association of a donor’s HCV positivity with time to post-transplant mortality and graft failure was evaluated by a Cox proportional hazard model with adjustment for clinical and demographic parameters of both donors’ and recipients’ and for immunosuppressant medications used in follow-up. *P*-values of 0.05 or lower were considered statistically significant. All analyses were run in SAS 9.3 (SAS Institute, Cary, NC).

For this study, a data use agreement with SRTR was signed, and a permission to publish the manuscript was obtained. The study was granted a non-human subject research exemption by Inova Institutional Review Board.

## Results

There were 106,427 liver transplants conducted between 1995 and 2013 included in SRTR. Of those, 95,710 were in individuals of at least 18 years of age, and in 75,469 of those the HCV status of a recipient, the HCV status of a donor, and a recipient’s mortality status were available. Of this subpopulation, 33,668 (44.6%) liver transplant recipients were HCV+.

**Fig. 1:**
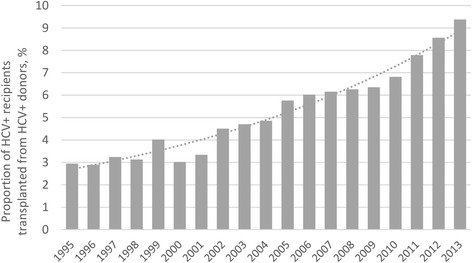
The proportion of HCV+ donors used for transplantation in HCV+ recipients by year

There was a substantial increase in the proportion of HCV+ recipients being transplanted from HCV+ donors during the study years. Indeed, the increase in less than 20 years was more than three-fold, from less than 3% in 1995 to more than 9% in 2013. The greatest change in the trend happened in 2010 which resulted in a nearly 50% increase in the proportion of HCV+ donors used for transplantation in the following 3 years, by at least 10% annually (Fig. 1).

Comparison of clinico-demographic parameters of HCV+ liver transplant recipients transplanted from HCV+ and HCV- donors is included in Table 1. There was a greater proportion of African-American patients receiving HCV+ transplants, as well as more patients with pre-transplant type 2 diabetes, liver cancer, and a greater proportion of re-transplants (all *p* < 0.05) (Table 1). Despite this, patients transplanted with HCV+ organs had lower MELD scores both with and without adjustment for the presence of liver cancer (both *p* < 0.0001).

**Table 1:** Comparison of HCV+ liver transplant recipients, donors and outcomes in transplantations from HCV+ and HCV-donors

	HCV+ donor	HCV- donor	*P*
*N*	1930	31,738	
Recipients:			
Age, years	55.1 ± 7.2	53.9 ± 7.7	<0.0001
Male gender	1448 (75.0%)	23,508 (74.1%)	0.35
Race/ethnicity: Caucasian	1346 (69.7%)	22,575 (71.1%)	0.19
Race/ethnicity: African-American	267 (13.8%)	3249 (10.2%)	<0.0001
Race/ethnicity: Hispanic	254 (13.2%)	4664 (14.7%)	0.06
Co-infected with HBV (HBV sAg+)	51 (2.7%)	959 (3.1%)	0.32
Pre-transplant history of type 2 diabetes	236 (13.3%)	3335 (11.7%)	0.0441
Liver cancer	544 (28.2%)	7414 (23.4%)	<0.0001
Liver re-transplant	3 (0.2%)	6 (0.0%)	0.0004
MELD score	18.2 ± 8.3	20.3 ± 9.7	<0.0001
MELD score excl. liver cancer	19.9 ± 8.3	22.4 ± 9.5	<0.0001
Donors:			
Age, years	42.0 ± 11.8	39.6 ± 16.4	<0.0001
Male gender	1196 (62.0%)	19,513 (61.5%)	0.67
Non-heart-beating	31 (1.6%)	1138 (3.6%)	<0.0001
History of diabetes	167 (8.7%)	2491 (7.9%)	0.19
History of cancer	39 (2.0%)	834 (2.6%)	0.11
History of high risk behavior	808 (42.4%)	2044 (6.5%)	<0.0001
Heterotopic transplant	2 (0.1%)	35 (0.1%)	0.95
Outcomes:			
Acute rejection episodes before discharge	36 (2.4%)	809 (4.1%)	0.0020
Discharged alive	1842 (95.4%)	29,808 (93.9%)	0.0063
Length of inpatient stay, days	15.7 ± 20.3	16.4 ± 21.9	0.0013
Mortality: 1 year	242 (12.5%)	4467 (14.1%)	0.059
Mortality: 3 years	399 (24.2%)	7190 (24.8%)	0.58
Mortality: 5 years	424 (33.0%)	8028 (32.5%)	0.68
Mortality: 10 years	281 (47.5%)	7019 (48.6%)	0.59
Graft failure: 1 year	38 (2.2%)	782 (2.8%)	0.15
Graft failure: 3 years	63 (4.8%)	1375 (5.9%)	0.09
Graft failure: 5 years	70 (7.5%)	1465 (8.1%)	0.56
Graft failure: 10 years	50 (13.9%)	1160 (13.5%)	0.86

The HCV+ donors were, on average, older, less frequently non-heart-beating at the time of procurement, and were substantially more frequently classified as high risk (all *p* < 0.0001). There was no difference in donors’ gender and history of diabetes or cancer (all *p* > 0.05) (Table 1).

The short-term outcomes of HCV+ liver transplant recipients were more favorable in those transplanted from HCV+ donors, including a lower rate of inpatient acute rejection events and inpatient mortality, and a shorter inpatient stay (all *p* < 0.007) (Table 1). However, when adjusted for the year of transplant, no difference in any of those outcomes was found (all *p* > 0.10).

**Fig. 2:**
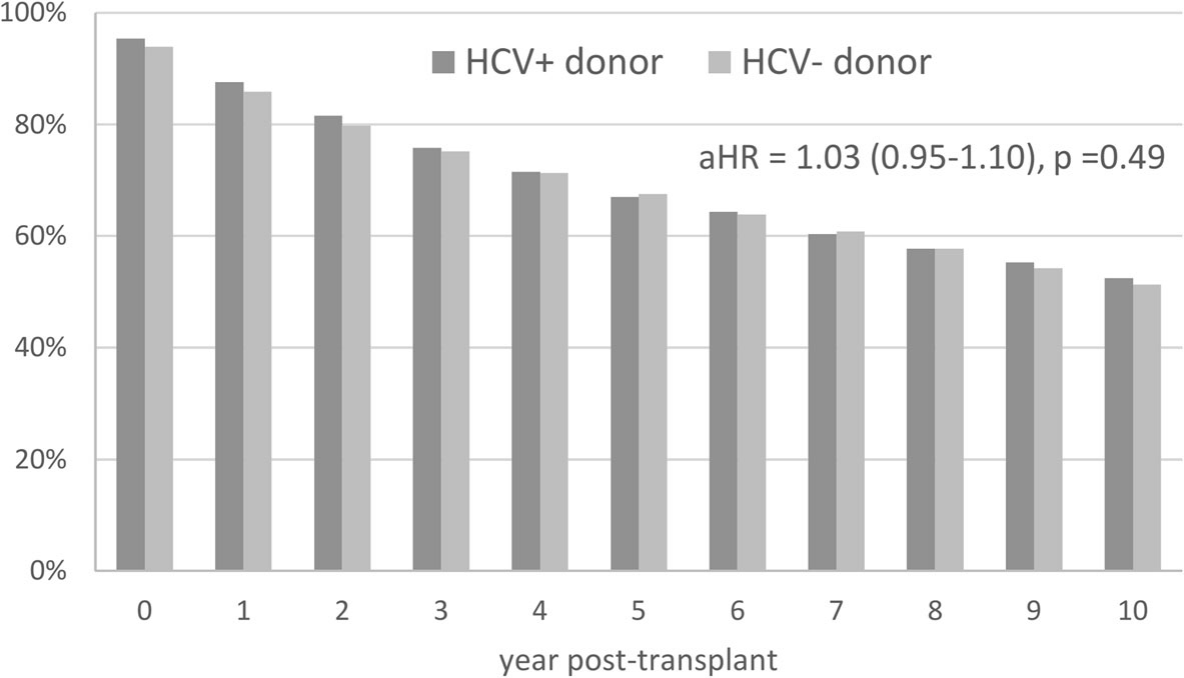
Post-transplant survival in HCV+ liver transplant recipients transplanted from HCV+ and HCV- donors. *P* > 0.05 at all time points. aHR – adjusted hazard ratio. Mortality at the zero time point represents inpatient mortality at transplant

Long-term outcomes were similar in patients transplanted from HCV+ and HCV- donors (Table 1, Fig. 2). Indeed, a borderline significant difference was observed for 1 and 2 years post-transplant mortality (1 year: 12.5% in patients transplanted from HCV+ donors vs. 14.1% in patients with HCV- donors, *p* = 0.059; 2 years: 18.4% vs. 20.2%, *p* = 0.054) which was again completely explained out by the bias in the year of transplant (both *p* > 0.21 after adjustment for year). At the same time, the following years after transplantation revealed nearly identical mortality rates (all *p* > 0.5).

Similarly, the graft loss rates were insignificantly lower in patients transplanted from HCV+ donors up to 3 years post-transplant (2.2% vs. 2.8% at 1 year, *p* = 0.15; 3.6% vs. 4.5% at 2 years, *p* = 0.090; 4.8% vs. 5.9% at 3 years, *p* = 0.089) due the yearly distribution of HCV+ donors (all *p* > 0.13 after adjustment for year).

In a Cox proportional hazard model, after adjustment for the year of transplant, recipient’s age, gender, race, elements of medical history, MELD score, donor’s age and medical history, and the use of immunosuppressant medications, the association of a donor’s HCV+ status with post-transplant graft loss and mortality in HCV+ liver transplant recipients was highly insignificant: adjusted hazard ratio (aHR) (95% CI) = 1.045 (0.954–1.145) for time to post-transplant mortality, aHR = 0.922 (0.727–1.169) for time to graft loss (both *p* > 0.33) (all mortality and graft loss predictors tested in the Cox proportional hazard models are included in Additional file 1: Table Table S1). Furthermore, the lack of association of donors’ HCV positivity with the studied outcomes was also confirmed in a separate round of case–control analysis where the outcomes of patients transplanted from HCV+ donors were compared to those of propensity score-matched controls (all *p* > 0.15) (Additional file 2: Table S2). In contrast, a more recent year of transplant was a major predictor of both lower mortality rate (aHR = 0.963 (0.956–0.971) per year) and lower graft failure rate (aHR = 0.950 (0.934–0.967) per year) (both *p* < 0.0001) even after adjustment for the type of immunosuppressants used in follow-up (Additional file 2: Table S2).

## Discussion

In this study, we reported post-transplant outcomes in patients with HCV transplanted from HCV antibody-positive donors and compared these outcomes to those of HCV patients transplanted from HCV-negative donors using 20 years of data from the nationwide transplant registry. Our study shows that there are no differences in both short- and long-term outcomes between patients who were transplanted from HCV-positive and HCV-negative donors. This is largely consistent with prior reports of similar findings reported from smaller studies with shorter follow-up.

This finding provides an additional support to the practice of using HCV-positive donors for HCV-positive recipients which has become nearly universal in multiple transplant centers. Indeed, we have shown that the use of HCV-positive organs has tripled over less than two decades. Nevertheless, our data confirm that the medium-term outcomes of patients with HCV are similar regardless of HCV positivity of the organs. Furthermore, the short-term outcomes, including being discharged alive from the hospital, having a rejection episode before discharge, and 1 year mortality, all seem to be lower in HCV-positive organ recipients. This superior short-term survival, in addition to the impact of a greater proportion of patients receiving HCV-positive organs in the most recent study years, may also be related to less advanced liver disease in these patients suggesting a cautious use of HCV-positive organs. Indeed, patients receiving HCV-positive organs had lower MELD scores which remained true even after excluding those who received MELD exclusion for hepatocellular carcinoma (HCC).

While there seems to be no evidence supporting the presence of an increased risk of post-transplant mortality and allograft losses arisen solely from the HCV-positivity of a donor, this finding should be interpreted with caution. In particular, it is necessary to understand that hepatocytes in an HCV-positive graft might still have been injured by chronic inflammation, so a careful selection of both grafts and recipients will be important [13]. Nevertheless, it is interesting to note that in our study, we have failed to find any evidence of an increased risk of adverse post-transplant outcomes associated with being HCV-positive in the group of donors age 45 or older or in the donors with history of type 2 diabetes (all *p* > 0.05).

With the new DAAs becoming more widely available, the utility of HCV-seropositive organs becomes even more important. Indeed, we suggest that the use of HCV-positive organs in HCV-positive recipients who can be safely and effectively treated post-transplant will become increasingly a viable option. However, it is important to note that although preliminary studies have shown notable benefits of treating post-transplant HCV patients with DAAs [14, 15], long-term data is still not available.

The study did have a limitation related to the lack of HCV RNA results for the donors, so the impact of active viremia in both donors and recipients could not be evaluated, while it is reasonable to believe that such impact may exist. We also did not have access to histology data for the grafts so the risk associated with the presence and/or degree of hepatic steatosis and fibrosis in an allograft could not be studied. The outcomes were limited to mortality and graft loss only, while other clinical outcomes, such as developing hepatic inflammation and fibrosis, could also significantly affect patients’ well-being in the post-transplant period.

## Conclusions

In this study which used the most recent nationwide registry of liver transplant recipients, we have found no evidence of an increased risk for adverse post-transplant outcomes that could be associated with HCV positivity of a donor. Therefore, we postulate that while we, as a society, are working on decreasing wait-list mortality in liver transplant candidates, HCV-positive allografts could be a reasonably safe option for patients with chronic HCV infection who are in need of a liver transplant. It is important to note that our data cannot be used in support of indiscriminate use of HCV-positive donors, especially those who are HCV RNA-positive, given previously reported unfavorable histologic outcomes which could not be ruled out in this study. Further studies are needed to establish evidence-based selection criteria for HCV-positive donors which could provide patients with the best possible risk-to-benefit ratio.

## Additional files

Additional file 1: Table S1. Independent predictors of post-discharge mortality and graft loss in patients with HCV. (DOCX 18 kb)
Additional file 2: Table S2. Case–control comparison of propensity score-matched HCV patients transplanted from HCV+ and HCV- donors. (DOCX 18 kb)

## Abbreviations

aHR: Adjusted hazard ratio
BMI: Body mass index
COPD: Chronic obstructive pulmonary disease
CVD: Cardiovascular disease
DAA: Direct-acting antiviral
HBV: Hepatitis B virus
HCC: Hepatocellular carcinoma
HCV: Hepatitis C virus
ICU: Intensive care unit
MELD: The model for end-stage liver disease
SRTR: The Scientific Registry of Transplant Recipients
