# Supplementary material for: Long-term outcomes of liver transplantation in patients with hepatitis C infection are not affected by HCV positivity of a donor
Source: BMC Gastroenterol. 2016 Nov 15;16:137. doi: 10.1186/s12876-016-0551-z (PMC5111255; doi:10.1186/s12876-016-0551-z)
Supplement: Additional file 1: Table S1. — Independent predictors of post-discharge mortality and graft loss in patients with HCV. (DOCX 18 kb) [file 12876_2016_551_MOESM1_ESM.docx]

**Table S1**. Independent predictors of post-discharge mortality and graft loss in patients with HCV.

|  | aHR | lower 95% CI | upper 95% CI | p |
| --- | --- | --- | --- | --- |
| **Time to death** |  |  |  |  |
| HCV+ donor | 1.045 | 0.954 | 1.145 | 0.34 |
| Calendar year, per year | 0.963 | 0.956 | 0.971 | <.0001 |
| Age, per year | 1.011 | 1.007 | 1.014 | <.0001 |
| Male gender (ref: female) | 0.917 | 0.871 | 0.965 | 0.0008 |
| African-American (ref: Caucasian) | 1.472 | 1.378 | 1.572 | <.0001 |
| Hispanic (ref: Caucasian) | 0.856 | 0.800 | 0.916 | <.0001 |
| History of type 2 diabetes | 1.272 | 1.191 | 1.358 | <.0001 |
| Liver cancer | 1.239 | 1.171 | 1.310 | <.0001 |
| Liver re-transplant | 1.128 | 0.363 | 3.500 | 0.83 |
| MELD score, per 1 point | 1.012 | 1.009 | 1.014 | <.0001 |
| Donor's age, per year | 1.017 | 1.016 | 1.019 | <.0001 |
| Donor after cardiac death | 1.168 | 1.040 | 1.312 | 0.0088 |
| Immunosuppressants: tacrolimus | 0.728 | 0.659 | 0.804 | <.0001 |
| Immunosuppressants: mycophenolates | 0.889 | 0.842 | 0.938 | <.0001 |
| Immunosuppressants: steroids | 1.109 | 1.010 | 1.218 | 0.0294 |
| Immunosuppressants: cyclosporine | 1.009 | 0.943 | 1.079 | 0.80 |
| **Time to graft loss** |  |  |  |  |
| HCV+ donor | 0.922 | 0.727 | 1.169 | 0.50 |
| Calendar year, per year | 0.950 | 0.934 | 0.967 | <.0001 |
| Age, per year | 0.981 | 0.974 | 0.989 | <.0001 |
| Male gender (ref: female) | 0.673 | 0.599 | 0.757 | <.0001 |
| African-American (ref: Caucasian) | 1.709 | 1.467 | 1.991 | <.0001 |
| Hispanic (ref: Caucasian) | 0.890 | 0.755 | 1.050 | 0.17 |
| History of type 2 diabetes | 1.017 | 0.852 | 1.215 | 0.85 |
| Liver cancer | 0.987 | 0.852 | 1.144 | 0.86 |
| Liver re-transplant | 0.000 | 0.000 | 1.11E+118 | 0.95 |
| MELD score, per 1 point | 1.017 | 1.011 | 1.024 | <.0001 |
| Donor's age, per year | 1.027 | 1.023 | 1.030 | <.0001 |
| Donor after cardiac death | 1.194 | 0.884 | 1.612 | 0.25 |
| Immunosuppressants: tacrolimus | 0.852 | 0.670 | 1.085 | 0.19 |
| Immunosuppressants: mycophenolates | 0.911 | 0.797 | 1.042 | 0.17 |
| Immunosuppressants: steroids | 1.108 | 0.878 | 1.399 | 0.39 |
| Immunosuppressants: cyclosporine | 1.215 | 1.042 | 1.416 | 0.0128 |
