# Supplementary material for: Long-term outcomes of liver transplantation in patients with hepatitis C infection are not affected by HCV positivity of a donor
Source: BMC Gastroenterol. 2016 Nov 15;16:137. doi: 10.1186/s12876-016-0551-z (PMC5111255; doi:10.1186/s12876-016-0551-z)
Supplement: Additional file 2: Table S2. — Case–control comparison of propensity score-matched HCV patients transplanted from HCV+ and HCV- donors. (DOCX 18 kb) [file 12876_2016_551_MOESM2_ESM.docx]

**Table S2**. Case-control comparison of propensity score-matched HCV patients transplanted from HCV+ and HCV- donors.

|  | HCV+ donor | HCV- donor | Prob |
| --- | --- | --- | --- |
| N | 1,736 | 1,736 |  |
| **Recipients:** |  |  |  |
| Age, years | 55.6 ± 6.9 | 55.6 ±7.2 | 0.83 |
| Male gender | 1,313 (75.6%) | 1,309 (75.4%) | 0.87 |
| Race/ethnicity: Caucasian | 1,197 (69.0%) | 1,181 (68.0%) | 0.56 |
| Race/ethnicity: African-American | 255 (14.7%) | 263 (15.1%) | 0.70 |
| Race/ethnicity: Hispanic | 227 (13.1%) | 223 (12.8%) | 0.84 |
| Co-infected with HBV (HBV sAg+) | 39 (2.3%) | 52 (3.1%) | 0.17 |
| Pre-transplant history of type 2 diabetes | 236 (14.7%) | 262 (16.1%) | 0.27 |
| Liver cancer | 525 (30.2%) | 526 (30.3%) | 0.97 |
| Liver re-transplant | 1 (0.1%) | 1 (0.1%) | 1.00 |
| MELD score | 18.2 ± 8.3 | 18.4 ± 9.0 | 0.70 |
| MELD score excl. liver cancer | 19.9 ± 8.3 | 20.6 ± 8.9 | 0.10 |
| **Donors:** |  |  |  |
| Age, years | 42.2 ± 12.0 | 42.3 ± 15.9 | 0.83 |
| Male gender | 1,070 (61.6%) | 1,038 (59.8%) | 0.27 |
| Non-heart-beating | 31 (1.8%) | 24 (1.4%) | 0.34 |
| History of diabetes | 159 (9.2%) | 156 (9.0%) | 0.83 |
| History of cancer | 38 (2.2%) | 54 (3.1%) | 0.10 |
| History of high risk behavior | 778 (45.3%) | 153 (8.8%) | <0.0001 |
| Heterotopic transplant | 2 (0.1%) | 1 (0.1%) | 0.56 |
| **Outcomes:** |  |  |  |
| Acute rejection episodes before discharge | 36 (2.4%) | 49 (3.3%) | 0.15 |
| Discharged alive | 1,662 (95.7%) | 1,658 (95.5%) | 0.74 |
| Length of inpatient stay, days | 15.3 ± 20.2 | 14.1 ± 16.4 | 0.39 |
| Mortality: 1 year | 213 (12.3%) | 238 (13.7%) | 0.21 |
| Mortality: 3 years | 353 (24.3%) | 347 (23.6%) | 0.67 |
| Mortality: 5 years | 365 (33.5%) | 350 (31.7%) | 0.37 |
| Mortality: 10 years | 191 (48.0%) | 187 (47.3%) | 0.86 |
| Graft failure: 1 year | 33 (2.1%) | 44 (2.9%) | 0.19 |
| Graft failure: 3 years | 54 (4.7%) | 61 (5.2%) | 0.59 |
| Graft failure: 5 years | 55 (7.1%) | 61 (7.5%) | 0.74 |
| Graft failure: 10 years | 30 (12.7%) | 33 (13.7%) | 0.74 |
